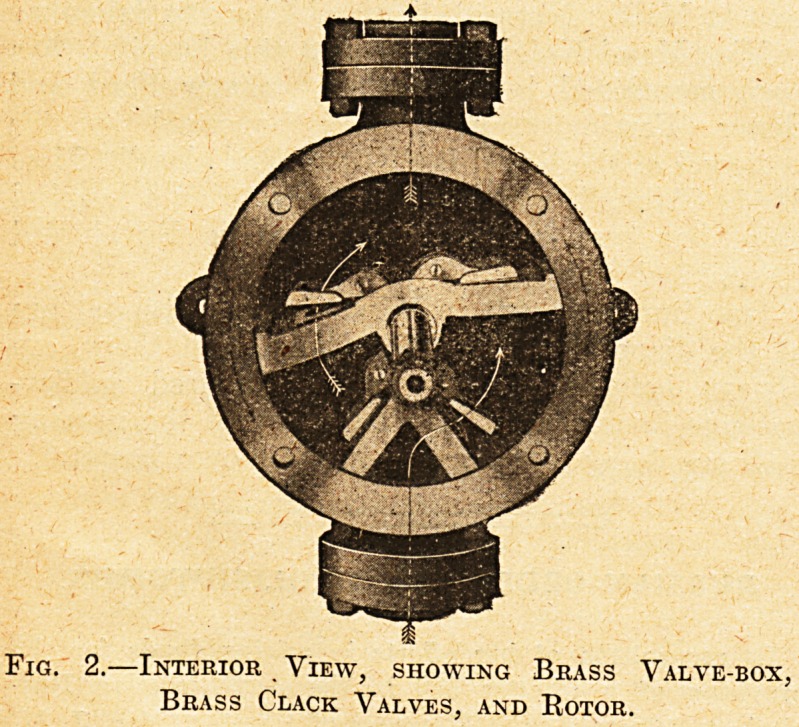# The "Runwell" Semi-Rotary Hand Pump

**Published:** 1917-03-31

**Authors:** 


### INSTITUTIONAL APPLIANCES.

### The "Runwell" Semi-Rotary Hand Pump.

A BRITISH FIRM CAPTURES A TRADE FROM THE GERMANS.

Messrs. Ashwell and Nesbit, of Leicester, who are well known as makers of heating, ventilating, and kindred apparatus, advise us that they have undertaken the manufacture of the above-named pump, which was imported into this country and the Colonies from Ger-

Fig. 1.

many prior to the war. Germany also exported the pump to the Latin countries. The firm have laid down special plant to manufacture these pumps, and report that they have several thousands on order for our own Government, and for our Allies and the Dominions. These pumps

Fig. 2.—Interior View, showing Brass Valve-box, Brass Clack Valves, and Rotor.

are as ingenious as they are simple. They are compact, effective, and deservedly popular wherever tried throughout the country. In a village in Wiltshire they are used for pumping water from an underground rain-water tank; they could be made equally available for pumping sewage or for filling water-carts from rivers or ponds, so that in gardens, households, on farms, and for agricultural purposes they fulfil many useful functions. A most ingenious use at petrol depôts is to attach one to the reservoir casks or tanks as a ready means of filling the spirit cans and other smaller vessels.

The pump is shown complete in fig. 1, and an inside view in fig. 2. It works upon the principle that has been developed very largely of late years, in which the suction and delivery is obtained by means of parts rotating inside the containing vessel, instead of moving to and fro. The pump is made in sizes from 7 inches over all, not including the handle, up to 2 feet, and for capacities ranging from 240 gallons per hour up to 7,500. Special forms of the pump are made for thick liquids and for hot liquids. Messrs. Ashwell and Nesbit have published their catalogue in English, French, Spanish, and Portuguese.

## Figures and Tables

**Fig. 1. f1:**
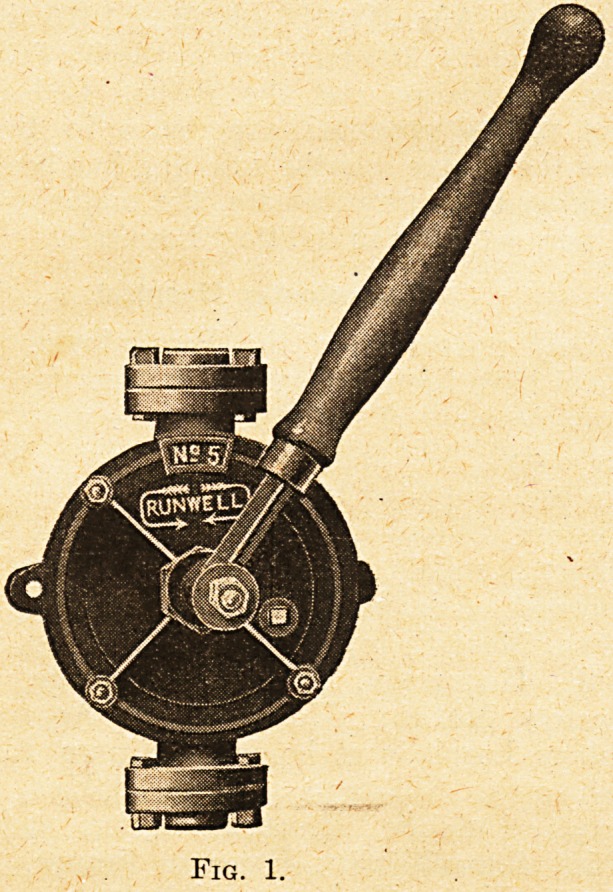


**Fig. 2. f2:**